# Induction of erythropoietin increases the cell proliferation rate in a hypoxia-inducible factor-1-dependent and -independent manner in renal cell carcinoma cell lines

**DOI:** 10.3892/ol.2013.1283

**Published:** 2013-04-03

**Authors:** YUTAKA FUJISUE, TAKATOSHI NAKAGAWA, KIYOSHI TAKAHARA, TERUO INAMOTO, SATOSHI KIYAMA, HARUHITO AZUMA, MICHIO ASAHI

**Affiliations:** 1 Departments of Urology, Faculty of Medicine, Osaka Medical College, Takatsuki, Osaka 569-8686, Japan; 2 Pharmacology, Faculty of Medicine, Osaka Medical College, Takatsuki, Osaka 569-8686, Japan

**Keywords:** erythropoietin, renal cell carcinoma, hypoxia-inducible factor-1

## Abstract

Erythropoietin (Epo) is a potent inducer of erythropoiesis that is mainly produced in the kidney. Epo is expressed not only in the normal kidney, but also in renal cell carcinomas (RCCs). The aim of the present study was to gain insights into the roles of Epo and its receptor (EpoR) in RCC cells. The study used two RCC cell lines, Caki-1 and SKRC44, in which Epo and EpoR are known to be highly expressed. The proliferation rate and expression level of hypoxia-inducible factor-1α (HIF-1α) were measured prior to and following Epo treatment and under normoxic and hypoxic conditions. To examine whether HIF-1α or Epo were involved in cellular proliferation during hypoxia, these proteins were knocked down using small interfering RNA (siRNA) in Caki-1 and SKRC44 cells. The results demonstrated that Epo enhanced the proliferation of the Caki-1 and SKRC44 cells. HIF-1α expression was increased upon the induction of hypoxia in the Caki-1 cells, but remained unaffected in the SKRC44 cells. The proliferation rate was increased under hypoxic conditions in the Caki-1 cells, but was decreased in the SKRC44 cells. Under hypoxic conditions, the proliferation of the Caki-1 cells was significantly reduced by the knock-down of HIF-1α or Epo, while the proliferation of the SKRC44 cells was significantly suppressed by the knock-down of Epo, but not HIF-1α. In conclusion, these data suggest that the induction of Epo may accelerate the proliferation of the RCC cell lines in either a HIF-1α-dependent or -independent manner.

## Introduction

Erythropoietin (Epo) is a 30-kDa glycoprotein that functions as an important cytokine in erythrocytes. Epo is usually produced by stromal cells of the adult kidney cortex or fetal liver and then released into the blood, with its production initially induced by hypoxia or hypotension (1–5). In the bone marrow, Epo binds to the erythropoietin receptor (EpoR) expressed in erythroid progenitor cells or undifferentiated erythroblasts, which induces signal transduction mechanisms that protect the undifferentiated erythrocytes from apoptosis and promote their proliferation and differentiation.

Previous studies have demonstrated that Epo and EpoR are not only produced or expressed in the kidneys, but also in other cells and tissues, including macrophages (6), vascular endothelial cells (7), neurons (8), myoblasts (9), the uterus, ovaries (10) and mammary glands (11), and in various malignant tumors, including angioblastomas (12), meningiomas (13), uterine or ovarian cancer (14) and breast cancer (15). The secretion of Epo due to hypoxia is upregulated by the induction of *Epo* mRNA via hypoxia-inducible factor (HIF)-1 signaling (16).

HIF-1 contains two subunits, HIF-1α and β. The expression of HIF-1α is altered by various conditions. HIF-1α is ubiquitinated and proteolyzed in the proteasome under normoxic conditions. With hypoxia, however, it is stabilized to bind with HIF-1β to form a heterodimer, which is internalized into the nucleus and binds with a hypoxia responsive element to induce Epo transcription. HIF-1β is constitutively expressed.

HIF-1 contributes to hypoxia adaptation at the tissue or cellular level. At the tissue level, it upregulates the expression levels of Epo and vascular endothelial growth factor (VEGF), which promote hematopoiesis and angiogenesis. At the cellular level, HIF-1 regulates cellular metabolism by the upregulation of glycolytic enzymes and glucose transporters, which shifts the energy metabolism to oxygen-independent glycolysis. Dysfunction in these pathways may be associated with the onset or progression of cancer (17), e.g., the progression of tumor angiogenesis by VEGF overexpression may be correlated with tumor infiltration or metastasis (18). Numerous anti-angiogenesis therapies against HIF-1 or VEGF have been developed and tested (19).

Epo binds to EpoR, resulting in the activation of the janus kinase-signal transducer and activator of transcription (JAK-STAT) signal transduction pathway (20). JAK2 phosphorylates STAT5, which is internalized into the nucleus to induce the transcription of specific genes (21). The proliferation signaling pathways, including the mitogen-activated protein kinase (MAPK) and phosphatidylinositol 3-kinase-Ak-thymoma (PI3K-AKT) pathways, which are involved in tumor proliferation, are also activated (22). A previous study demonstrated that EpoR was expressed in kidney tissues removed by nephrectomy or in renal cell carcinoma (RCC) cell lines, and that the proliferation rate of these cells increased with the addition of Epo in a dose-dependent manner (23). Furthermore, RCC patients with high expression levels of EpoR in the kidney tissues and high serum Epo (s-Epo) concentrations (>30 mU/ml) had better 5-year survival rates than those with low EpoR levels and s-Epo concentrations (24).

In the present study, five RCC cell lines were characterized and two cell lines in which Epo and EpoR were highly expressed were identified. The comparative study of these two cell lines revealed that the induction of Epo may accelerate cellular proliferation in either a HIF-1α-dependent or -independent manner. The possibility of using Epo as a promising target for anti-RCC drugs was also addressed.

## Material and methods

### Materials

The monoclonal anti-HIF-1α antibody was purchased from Invitrogen (Carlsbad, CA, USA) and the anti-Epo antibody was purchased from Santa Cruz Biotechnology, Inc. (Santa Cruz, CA, USA). The siRNAs against HIF-1α or Epo were purchased from Sigma-Aldrich (St. Louis, MO, USA).

### Cell culture

The 769P (renal cell adenocarcinoma), 786O (renal cell adenocarcinoma) and Ku19/20 cells (RCC) were maintained in RPMI-1640. The SKRC44 cells (RCC) were maintained in Dulbecco’s modified Eagle’s medium (DMEM) and the Caki-1 cells (RCC) were grown in minimal essential medium (MEM) in a humidified atmosphere containing 5% CO_2_ in the air (normoxia) (25). Unless stated otherwise, the media were supplemented with 10% fetal bovine serum (FBS), 100 U/ml penicillin and 100 *μ*g/ml streptomycin. The study was approved by the Ethics Committee of Osaka Medical College, Takatsuki, Osaka, Japan (569-0801).

### Real-time PCR

Total RNA was prepared from each of the five cell lines using TRIzol reagent (Invitrogen), according to the manufacturer’s instructions. Real-time PCR analyses were performed using a Thermal Cycler Dice Real Time System (Takara Bio, Shiga, Japan). The cDNA was synthesized using Superscript III reverse transcriptase (Invitrogen), according to the manufacturer’s instructions. Each reaction was performed in a total volume of 25 *μ*l, with 1× SYBR Premix Ex Taq polymerase, 200 nM primers, 2 *μ*l of a 1:10 dilution of the cDNA and RNase-free water. The thermal cycling conditions for the real-time PCR were; 10 sec at 95°C to activate the SYBR Ex Taq polymerase, followed by 40 cycles of denaturation for 5 sec at 95°C and annealing/extension for 20 sec at 60°C. The mean number of cycles required to reach the threshold of fluorescence detection was calculated for each sample and glyceraldehyde-3-phosphate dehydrogenase (*GAPDH*) expression was quantified for normalization of the amount of cDNA in each sample. The specificity of the amplified product was monitored using its melting curve. The primers used in the present study are as follows: *Epo*, forward primer, 5′-CCCTGTTGGTCAACTCTTCC-3′ and reverse primer, 5′-GTGTACAGCTTCAGCTTTCC-3′; *EpoR*, forward primer, 5′-GCACCGAGTGTGTGCTGAGCAA-3′ and reverse primer, 5′-GGTCAGCAGCACCAGGATGAC-3′; *GAPDH*, forward primer, 5′-ATTGCCCTCAACGACCACTT-3′ and reverse primer, 5′-AGGTCCACCACCCTGTTGCT-3′ (26,27)

### Cell proliferation assay [water-soluble tetrazolium salt-1 (WST-1) assay]

Cell proliferation under normoxic or hypoxic conditions was measured using Cell Counting Kit-8 (Wako Pure Chemicals, Osaka, Japan). Briefly, the cells were seeded at a density of 1×10^4^ per well in 96-well plates and allowed to attach overnight in appropriate serum-containing media. The cells were rendered quiescent by starvation in serum-free media for 24 h. FBS was used as a positive control at 10% of the total media volume. Various Epo doses (0.1, 0.33, 1, 3.3 and 10 ng/ml) were then added and incubation was continued for a further 24 h (28). The cellular proliferation was measured by adding a 1/10 volume of WST-1. Following a 1-h incubation with WST-1, the absorbance at 450 nm was recorded using a plate reader (Bio-Rad, Hercules, CA, USA).

### HIF-1α induction

For HIF-1α induction, the cells were incubated with 125 *μ*M of cobalt chloride (CoCl_2_) or were cultured in a hypoxia chamber (Stemcell Technologies, Vancouver, Canada), which was adjusted to 5% O_2_ and 5% CO_2_ for the period of interest.

### Western blot analysis

The cells were harvested in lysis buffer containing 20 mM Tris-HCl (pH 7.4), 150 mM NaCl, 5 mM ethylenediaminetetraacetic acid (EDTA), 1% (w/v) Nonidet P-40, 10% (w/v) glycerol, 5 mM sodium pyrophosphate, 10 mM sodium fluoride, 1 mM sodium orthovanadate, 10 mM β-glycerophosphate, 1 mM phenylmethylsulfonyl fluoride, 2 *μ*g/ml aprotinin, 5 *μ*g/ml leupeptin, and 1 mM dithiothreitol. In total, five micrograms of the cellular proteins (50 *μ*g for Epo detection) were electrophoresed and blotted onto a polyvinylidene difluouride (PVDF) membrane (FluoroTrans, Pall, Pensacola, FL, USA). Subsequent to blocking with 5% skimmed milk in Tris-buffered saline (TBS; 25 mM Tris, 0.15 M NaCl, pH 7.4) containing 0.1% Tween-20 (TBS-T) for 1 h, the membrane was incubated with a 1:1000 dilution of each antibody for a further hour. The membrane was washed three times with TBS-T for 10 min each, then the second antibody was added at a dilution of 1:2500. Following another three washes with TBS-T for 10 min each, the membrane was developed with Immobilon Western developer (Merck Millipore, Billerica, MA, USA) according to the manufacturer’s instructions. The image was captured using a ChemiDoc imaging system (Bio-Rad).

### Knock-down of HIF-1α or Epo using siRNA in Caki-1 and SKRC44 cells

The siRNA was transfected into Caki-1 and SKRC44 cells using Lipofectamine 2000 (Invitrogen), following the manufacturer’s instructions. Briefly, 50 pmol siRNA in 100 *μ*l Opti-MEM was mixed with 3 *μ*l of Lipofectamine 2000 diluted in 100 *μ*l Opti-MEM. The mixture was allowed to stand for 20 min and then was added to the cultured cells to incubate overnight in 1 ml antibiotic-free medium in a 12-well plate. After 48 h, the cells were utilized for the cell proliferation assays and in the determination of the *HIF-1*α and *Epo* mRNA expression levels.

### Statistical analysis

For comparisons between the two groups, the paired Student’s t-test was used to compare the significance of the differences between data. When more than two groups were compared, a one-way analysis of variance (ANOVA) was used followed by multiple comparisons using Dunnett’s test. P<0.05 was considered to indicate a significant difference. All data are expressed as the mean ± standard deviation (SD).

## Results

### Characterization of RCC cell lines

**Figure 1:**
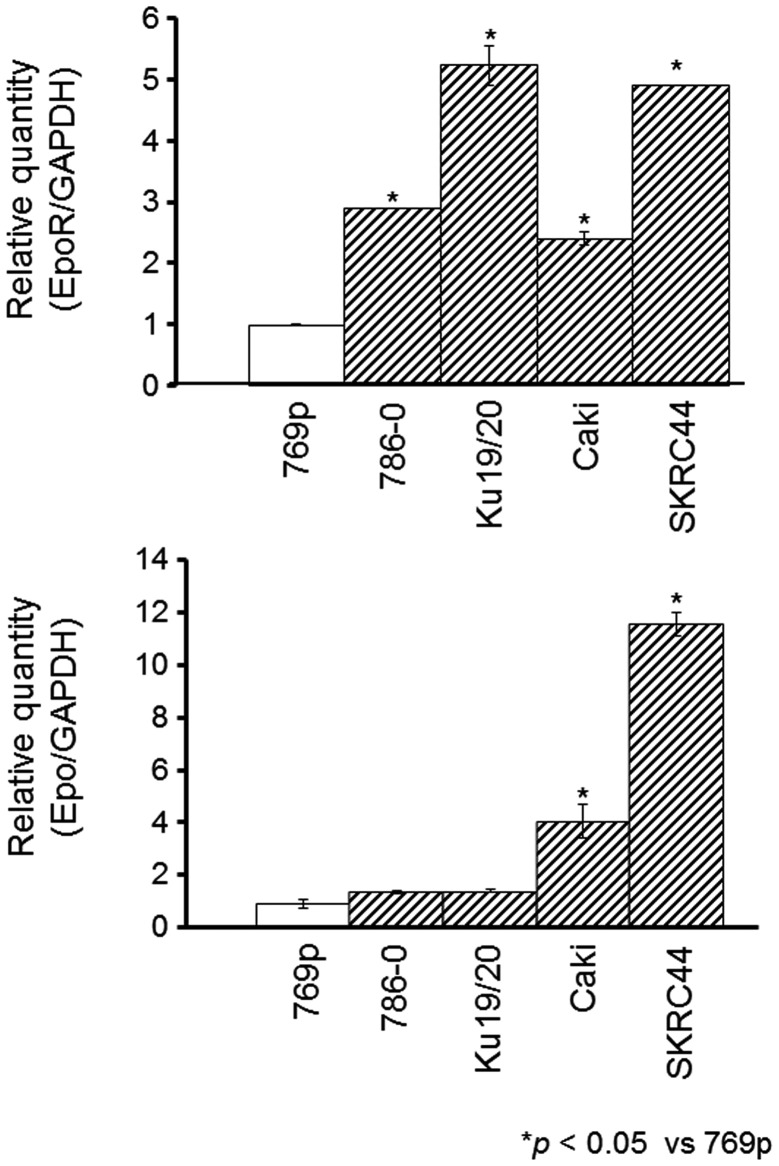
Real-time PCR measurements of erythropoietin (*Epo*) and Epo receptor (*EpoR*) expression in renal cell carcinoma (RCC) cell lines. A total of five cell lines were used; 769P, 786O, Ku19/20, Caki-1 and SKRC44. Total RNA was extracted and analyzed for *Epo* and *EpoR* expression by real-time PCR. Caki-1 and SKRC44 cells highly express both Epo and EpoR. Glyceraldehyde-3-phosphate dehydrogenase (*GAPDH*) was used as the housekeeping control gene.

**Figure 2:**
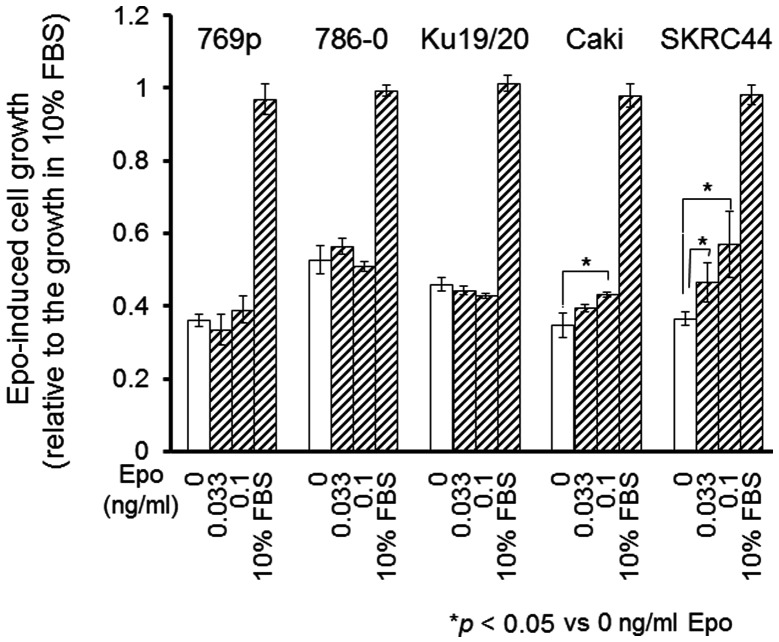
Erythropoietin (EPO)-induced cell growth in renal cell carcinoma (RCC) cell lines. Proliferation of 769P, 786O, Ku19/20, Caki-1 and SKRC44 cells in the presence of fetal bovine serum (FBS; 10%) or exogenous erythropoietin (0, 0.033 and 0.1 ng/ml) is shown in the bar graph. Caki-1 and SKRC44 cells highly express Epo. The proliferation rate of each cell line was measured using the water-soluble tetrazolium salt-1 (WST-1) assay as described in the Materials and methods.

The mRNA expression levels of *Epo* and its receptor, *EpoR*, were evaluated in five RCC cell lines; 769p, 786O, Ku19/20, Caki-1 and SKRC44. Real-time PCR confirmed that the *Epo* expression was significantly higher in the 786O, Ku19/20, Caki-1 and SKRC44 cells, compared to the 769P cells. In contrast, the *Epo-R* expression was significantly higher in the Caki-1 and SKRC44 cells, but was unchanged in the 786O and Ku19/20 cells (Fig. 1). Next, following the addition of the Epo protein, cellular proliferation was analyzed using the WST-1 cell proliferation assay. Of note was the fact that the SKRC44 and Caki-1 cells grew more rapidly in the presence of Epo, while the proliferation of the other cells remained unchanged by the addition of Epo (Fig. 2).

### Effects of CoCl_2_ treatment or hypoxia on the induction of HIF-1α protein expression in Caki-1 and SKRC44 cells

**Figure 3:**
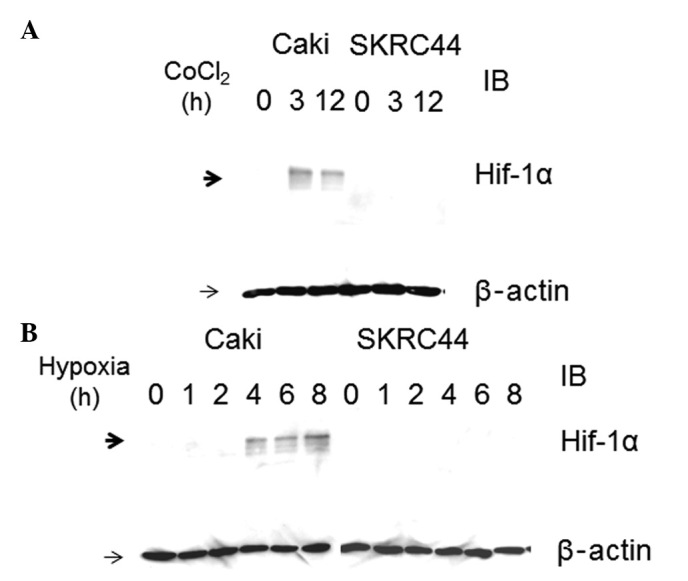
Cobalt chloride (CoCl_2_)- or hypoxia-induced hypoxia inducible factor (HIF)-1α expression in Caki-1 and SKRC44 cells (A) For HIF-1α induction, the cells were incubated with 125 *μ*M CoCl_2_. HIF-1α and β-actin protein levels were detected by western blot analysis of whole-cell extracts, as described in the Materials and methods, and were measured at 0, 3 and 12 h subsequent to incubation. (B) Caki-1 and SKRC44 cells were exposed to hypoxia for 8 h and measured at 0, 1, 2, 4, 6 and 8 h. Hypoxia induces Hif-1α expression in Caki-1 cells, but not in SKRC44 cells. HIF-1α and β-actin protein levels were detected by western blot analysis of whole-cell extracts, as described in the Materials and methods. IB, immunoblot.

Since it was recently reported that RCC patients with high expression levels of Epo and EpoR have a poor prognosis (24), we selected Caki-1 and SKRC44 cells, the two cell lines in which Epo and EpoR are highly expressed, for further characterization of the mechanisms responsible for this prognosis. As Epo expression is regulated by HIF-1α, the expression level of the HIF-1α protein was evaluated in each cell line. To induce HIF-1α, CoCl_2_, which is known to stabilize HIF-1α, was first exploited by interfering with its prolyl hydroxylation. HIF-1α was induced by CoCl_2_ treatment in the Caki-1 cells in a time-dependent manner. In contrast, the HIF-1α protein expression was not increased in the SKRC44 cells, even following 12-h incubations with CoCl_2_ (Fig. 3A).

To further confirm the HIF-1α expression patterns in the Caki-1 and SKRC44 cells, the cells were cultured under hypoxic conditions for 0, 1, 2, 4, 6 and 8 h. HIF-1α protein was induced under hypoxic conditions, as well as with CoCl_2_ treatment, in the Caki-1 cells, but not in the SKRC44 cells (Fig. 3B). As shown in Fig. 1B, the *Epo* mRNA was highly expressed in the Caki-1 and SKRC44 cells. Despite the high expression level of *Epo*, HIF-1α protein expression was not induced following CoCl_2_ treatment or with hypoxic conditions in the SKRC44 cells.

### Effects of hypoxia on cell proliferation in Caki-1 and SKRC44 cells

**Figure 4:**
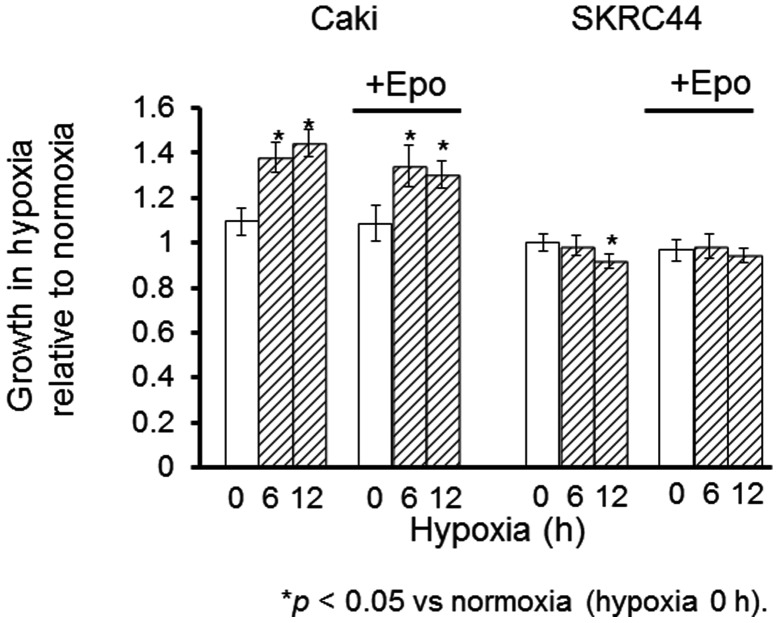
Effects of hypoxia on the proliferation of Caki-1 and SKRC44 cells. The proliferation rate was measured at 0, 6 and 12 h subsequent to incubation with exogenous erythropoietin (Epo) or control for 12 h under hypoxia. Caki-1 cells that express Hif-1α in response to hypoxia grow in a hypoxic condition. Growth of SKRC44 cells that do not express Hif-1α in hypoxia is suppressed in a hypoxic condition, but the suppression is relieved by adding exogenous Epo. The bar graph shows the mean proliferation rate and the error bars indicate the standard error.

Tthe proliferation rates were measured in the Caki-1 and SKRC44 cells under hypoxic conditions for 6 or 12 h with or without exogenous Epo (0.1 ng/ml). Epo stimulated the proliferation of the Caki-1 cells under normoxia; this increase was maintained under hypoxia. In contrast, the proliferation rate of the SKRC44 cells was not changed by the addition of Epo under hypoxia. Instead, this rate was significantly decreased under hypoxia for 12 h relative to the rate at normoxia. Exogenous Epo reversed the suppression of proliferation in the SKRC44 cells under hypoxic conditions (Fig. 4).

### Effects of Epo or HIF-1α silencing on the proliferation of SKRC44 and Caki-1 cells

The proliferation rate was significantly increased under hypoxia in the SKRC44 and Caki-1 cells. To elucidate whether HIF-1α or Epo was involved in this increased proliferation rate under normoxia or hypoxia in the cells, HIF-1α or Epo were knocked down using siRNA. It is well-known that HIF-1α induces the expression of various molecules associated with cell proliferation, including Epo. Epo expression was significantly decreased with the use of siRNA against Epo, but not with siRNA against HIF-1α in the SKRC44 cells, while the Epo expression was significantly decreased with the siRNAs against Epo and HIF-1α in the Caki-1 cells (data not shown).

**Figure 5:**
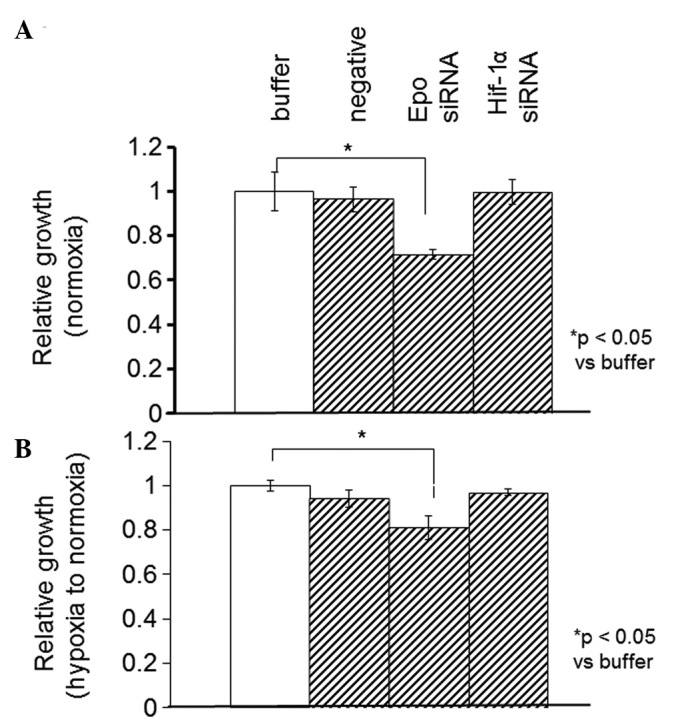
Effects of siRNAs against HIF-1α or erythropoietin (Epo) on cell proliferation under (A) normoxia or (B) hypoxia in SKRC44 cells. SKRC44 cells were treated overnight with buffer control, negative control and siRNAs against HIF-1α or Epo, and cultured for 48 h. The cells were cultured for an 12 h under normoxia or hypoxia. SKRC44 cells treated with buffer control under normoxia were used as control. An siRNA against Epo inhibited growth of SKRC44 in both hypoxia and normoxia. An siRNA against Hif-1α did not inhibit growth of SKRC44. The proliferation rate was measured using the WST-1 assay. HIF, hypoxia-inducible factor; siRNA, small interfering RNA.

**Figure 6:**
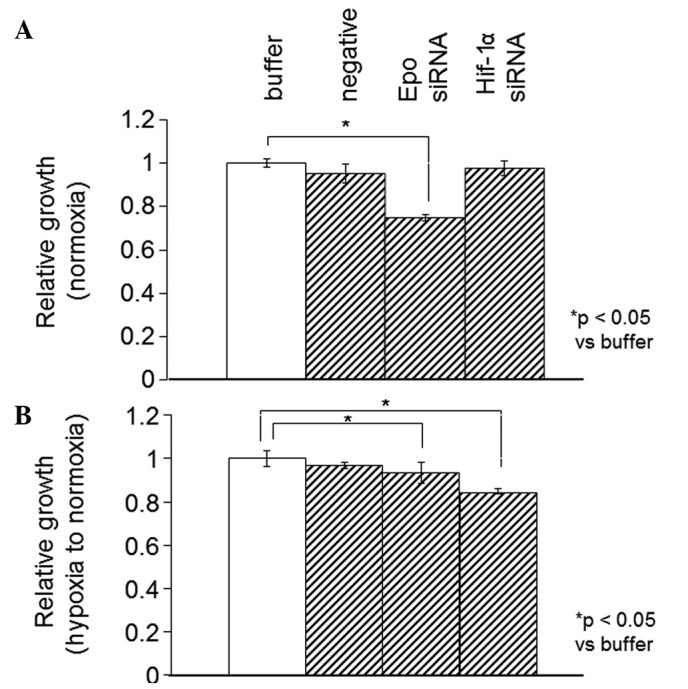
Effects of siRNA against HIF-1α or erythropoietin (Epo) on cell proliferation under (A) normoxia or (B) hypoxia in Caki-1 cells. Caki-1 cells were treated overnight with buffer control, negative control and siRNAs against HIF-1α or Epo, and then cultured for 48 h. The cells were cultured for an additional 12 h under normoxia or hypoxia. Caki-1 cells treated with buffer control under normoxia were used as a control. An siRNA against Epo inhibited growth of Caki-1 cells. In contrast, an siRNA against Hif-1α inhibited growth of Caki-1. The proliferation rate was measured using the WST-1 assay. HIF, hypoxia-inducible factor; siRNA, small interfering RNA.

Using the WST-1 assay, the proliferation rate was measured in the SKRC44 or Caki-1 cells 12 h after the addition of the control buffer (10 mM Tris, 20 mM NaCl and 1 mM EDTA; pH 8.0), negative siRNA (Mission siRNA Universal Negative Controls, Sigma-Aldrich) or siRNAs against Epo or HIF-1α under normoxia or hypoxia. In the SKRC44 cells, the proliferation rate when using the siRNA against Epo was significantly decreased compared with the rate following the addition of the control buffer under both normoxic and hypoxic conditions. The proliferation rate was unchanged by the siRNA against HIF-1α (Fig. 5). In the Caki-1 cells, the Epo expression level was significantly decreased by the siRNAs against Epo and HIF-1α (data not shown). The WST-1 proliferation assay was also performed 12 h subsequent to the same treatments, as described previously for the SKRC44 cells under normoxia or for hypoxia in the Caki-1 cells. In the Caki-1 cells, the proliferation rate when using the siRNA against Epo was significantly decreased under normoxia and hypoxia. Under hypoxia, however, this rate was significantly decreased with the siRNAs against Epo and HIF-1α (Fig. 6).

## Discussion

It has been previously demonstrated that EpoR, the receptor through which Epo stimulates mitogenesis, is expressed in human RCC tissue and cell lines (23). The co-expression of Epo and EpoR has been detected in numerous renal cysts, providing further evidence that renal cysts are potential precursors to RCC. In conjunction with von Hippel-Lindau (VHL) gene deficiency, the co-expression of Epo and EpoR in renal cysts and tumors may reflect developmental arrest in immature mesenchymal cells. Such arrest may lead to autocrine stimulation, cellular proliferation and renal tumor development (26).

In the present study, Epo and EpoR were each identified to be highly expressed in Caki-1 and SKRC44 cells, and the proliferation rate in these two cell lines was observed to be increased in the presence of Epo.

The expression of Epo in RCC and renal cysts may result from VHL gene deficiency through the HIF-1 pathway (27,28). EpoR expression normally occurs during the angioblast stage of embryonic development as part of the response to hypoxia. During normal development, cellular EpoR expression is transient (29). Continuous high expression of Epo and EpoR in primitive mesenchymal cells may lead to cellular proliferation via autocrine stimulation and become a critical pathogenic step in tumor formation (30). Therefore, the co-expression of Epo with EpoR in VHL-associated RCC and renal cysts suggests that a precursor cell of renal lesions is a developmentally arrested, pluripotent embryonic cell derived from nephrogenous mesenchyme (26).

HIF-1α is a well-known transcriptional inducer of survival proteins, such as Epo (31). Epo, a downstream protein of HIF-1α, has been observed to counteract the hypoxia-induced apoptosis of breast cancer cells (32). This association was demonstrated by the correlations between the degrees of immunohistochemical expression of HIF-1 and Epo in previous studies (33).

To examine whether HIF-1α is involved in the expression of Epo, the HIF-1α expression levels in the Caki-1 and SKRC44 cells were determined by western blot analyses. HIF-1α protein expression was increased under hypoxic conditions in the Caki-1 cells, but not in the SKRC44 cells (Fig. 3); although the expression level of Epo and EpoR in normoxic conditions was higher in the SKRC44 cells compared with the Caki-1 cells (Fig. 1). These observations indicate that Epo and EpoR expression may not be induced by HIF-1α in SKRC44 cells. As Caki-1 and SKRC44 cells are each derived from clear cell carcinomas, the high Epo and EpoR expression levels may be either dependent or independent of HIF-1α in clear cell carcinoma cell lines.

The overexpression of HIF-1α has been reported in numerous human cancers, including colon, brain, breast, gastric, lung, skin, ovarian, prostate, renal and pancreatic carcinoma, and is associated with a poor prognosis and failure of tumor treatment (34). In tumor cells, HIF-1α may also be regulated by other genetic factors, including oncogenes (*Ras* and *PI3-K*) or the loss of tumor suppressors [*VHL* or phosphatase and tensin homolog (*PTEN*)] even under aerobic conditions. Therefore, the inhibition of HIF-1α may represent an attractive strategy with synergistic potential when used with other therapies (35,36).

We next measured the proliferation rates in the Caki-1 and SKRC44 cells under hypoxia with or without exogenous Epo (Fig. 4). The proliferation rate was significantly increased under hypoxia in the Caki-1 cells in which HIF-1α was inducible, while it was decreased in the SKRC44 cells in which HIF-1α expression was not increased. To elucidate the involvement of HIF-1α in increasing the proliferation of the Caki-1 and SKRC44 cells under hypoxic conditions, HIF-1α was knocked out using siRNA. The proliferation rate of the Caki-1 cells was significantly decreased by the siRNA against HIF-1α, although it remained unchanged in the SKRC44 cells (Figs. 5 and 6). These results indicated that HIF-1α may be partially implicated in the progression of RCC.

Epo expression levels are regulated by pVHL, which has been shown to control the stability of HIF-1 through ubiquitination and proteasomal degradation mechanisms (37,38). Upregulated HIF-1α stimulates Epo expression in tumors, indicating that the VHL-HIF-Epo pathway may be significant in controlling the proliferation of RCC cells (39). The present study demonstrated that Epo expression was independent of this pathway; therefore, blocking this pathway alone may not be sufficient to inhibit Epo expression. Thus, siRNA against Epo was transfected into the Caki-1 and SKRC44 cells. The proliferation of the two cell lines was significantly decreased by the siRNA against Epo (Figs. 5 and 6). This observation indicated that Epo may be implicated in the progression of RCC. Since Caki-1 and SKRC44 cells are RCC-derived cells, HIF-dependent and-independent Epo expression may control the proliferation of RCC cells. Regardless of the mechanism by which HIF-1α is expressed, the RCC progression rate under normoxia and hypoxia could be reduced by modulating the expression level of Epo.

Recently, a number of molecular target based drugs have been developed for the treatment of RCC. Tyrosine kinase inhibitors, including sunitinib and sorafenib, are representative of such agents that inhibit the activity of VEGF-mediated signal transduction in cancer cells. Everolimus, an inhibitor of the mammalian target of rapamycin (mTOR), has been shown to be effective for the treatment of advanced RCC following treatment failure with the first-line drugs sunitinib or sorafenib. Everolimus reduces cellular proliferation, angiogenesis and glucose uptake via the inhibition of HIF-1α expression, which upregulates VEGF-mediated signal transduction in cancer cells (40,41).

In the present study, we demonstrated that the induction of Epo in a HIF-1-dependent and -independent manner increases the cellular proliferation rate in RCC cell lines. Notably, it has been reported that the proliferation rate of the RCC cells with HIF-1-independent Epo overexpression was not fully reduced by everolimus. Elucidation of the mechanism of HIF-1-independent Epo induction in RCC may lead to the identification of a new molecular target candidate for RCC therapy, particularly in clear cell carcinoma. Although further study is required to identify the involvement of the HIF-Epo pathway in RCC pathogenesis, the pathway may be a new molecular therapeutic target candidate for the treatment of RCC, particularly in advanced stages.

## Abbreviations:

Epo: erythropoietin;
EpoR: erythropoietin receptor;
RCC: renal cell carcinoma;
HIF-1α: hypoxia-inducible factor-1α;
VHL: von Hippel-Lindau
